# Dental aerosols: microbial composition and spatial distribution

**DOI:** 10.1080/20002297.2020.1762040

**Published:** 2020-05-13

**Authors:** C. Zemouri, C.M.C. Volgenant, M.J. Buijs, W. Crielaard, N.A.M. Rosema, B.W. Brandt, A.M.G.A. Laheij, J.J. De Soet

**Affiliations:** a Department of Preventive Dentistry, Academic Centre for Dentistry Amsterdam, University of Amsterdam and Vrije Universiteit Amsterdam, Amsterdam, The Netherlands; b Department of Periodontology, Academic Centre for Dentistry Amsterdam, University of Amsterdam and Vrije Universiteit Amsterdam, Amsterdam, The Netherlands

**Keywords:** Dentistry, dental clinic, bio-aerosol, microbiology, infection control

## Abstract

**Background**: High-speed dental instruments produce aerosols, which can contribute to the transmission of pathogenic microorganisms. The aim of this study is to describe the microbial load and – composition and spatial distribution of aerosols in dental clinics.

**Methods**: In four dental clinics active and passive sampling methods were used before, during and after treatment and at different locations. Retrieved colony forming units (CFU) were sequenced for taxon identification.

**Results**: The samples contained up to 655 CFU/plate/30 minutes and 418 CFU/m^3^/30 minutes during dental treatment for active and passive sampling, respectively. The level of contamination after treatment and at 1.5 m distance from the patient’s head was similar to the start of the day. The highest contamination was found at the patient’s chest area. The aerosols consisted of 52 different taxa from human origin and 36 from water.

**Conclusion**: Contamination in dental clinics due to aerosols is mainly low, although high level of contamination with taxa from both human and water origin was found within 80 cm around the head of the patient. Our results stress the importance of infection control measures on surfaces in close proximity to the head of the patient as well as in dental water lines.

## Introduction

In dental clinics, aerosols are produced while using dental hand pieces, such as ultrasonic scalers, air rotors, micromotors and/or air-water syringes [1]. These bio-aerosols consist of droplets, also known as spatters, and droplet nuclei [2,3]. Droplets, sized >100 μm, settle rapidly onto surfaces in the immediate proximity of the source. Droplet nuclei, <10 μm, are lighter and, therefore, can remain in the air for hours before settling on(to) a surface [2]. These aerosols contain microorganisms originating from the patient’s oral cavity and from dental unit waterlines (DUWLs) [4]. The release of microorganisms into aerosols increases the microbial burden in the air and can lead to the contamination of all surfaces in a dental treatment room. Because of the frequent aerosol generating procedures in dental practice, these aerosols can function as an important mode for infection transmission in dental clinics [4].

Since dental instruments are the generating source of aerosols, the microbial contamination of DUWLs plays an important role. The biofilm in DUWLs may harbour opportunistic pathogens, such as *Pseudomonas* spp. and *Legionella pneumophila*, while both species have been found in air samples [4–7]. In addition, DUWL water with a high load of Gram-negative bacteria harbours endotoxins, which can lead to respiratory complaints when inhaled [5,8,9]. Oral microorganisms, such as *Micrococcus* spp. and *Corynebacteria* spp., were also found in aerosols indicating the presence of oral microorganisms in the droplets and droplet nuclei [8,10–12]. Settled aerosols, containing microorganisms from water and the oral cavity, are likely to carry infectious microorganisms and may lead to cross-transmission and infection in susceptible patients and dental staff [2,13,14]. However, evidence on the microbial characteristics of aerosols in dental clinics is limited [15]. So far, only 19 bacterial species were reported to be present in the aerosols around the patient, wherefrom most were *Staphylococcus* spp. The spatial distribution of aerosols is reported in cross-sectional studies [15]. Yet, results and conclusions differ between studies. This might be due to different sampling methods, sampling strategies and differences in culturing the microorganisms [15].

Aerosol formation in dental clinics is unavoidable [4], yet the release of water and oral microorganisms into the generated aerosols increase the risk of cross-contamination. Therefore, the patients and dental healthcare workers are at risk for acquiring infections [8,9,16]. The present study aimed to quantify the spatial distribution of aerosols in dental clinics as well as its microbial load and composition. The presented findings increase the awareness into the risks of cross-contamination in dental clinics and could have direct implications on infection control measures.

## Materials and methods

### Ethical approval

Air was sampled before, during and after patient treatment in four dental clinics in The Netherlands; three dental private clinics (referred to as clinic 1, 2, and 3) and a treatment room at a university dental treatment centre (clinic 4). The Institutional Review Board of ACTA approved the study protocol (reference number 2,018,024).

### Air sampling

**Figure 1.**
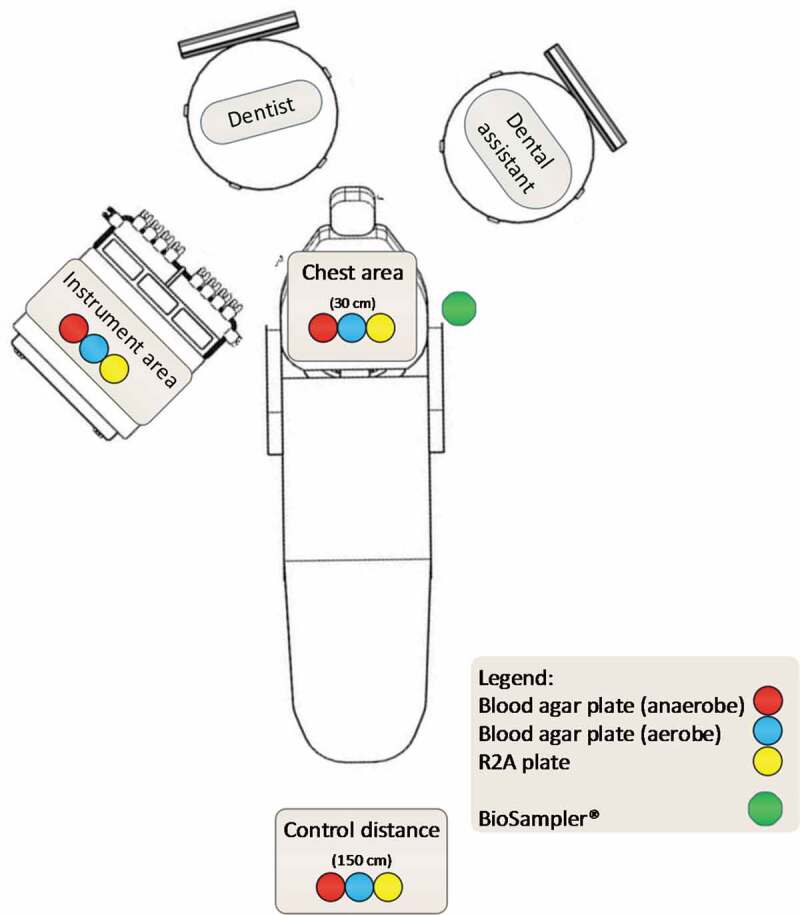
Floor plan of the four dental clinics with the placement of agar plates during treatment (passive sampling) and the location of the BioSampler® during active sampling. Passive sampling an active sampling was not performed on the same day. The dental assistant was present in clinic 1 and 3

The microbial load in the air of the dental clinics was measured using passive and active sampling methods. Passive sampling was conducted by exposing 90 mm diameter petri dishes containing either blood agar (Colombia blood agar base (Hach, Loveland, USA), supplemented with 5% sheep blood) or R2A agar (Hach) to the air for 30 minutes at 80 cm height from the floor. The air was sampled at three moments during a normal day of patient treatment: 1) 30 minutes before the first treatment. The room was unoccupied for at least 12 hours; 2) during the dental treatment; and 3) 30 minutes after the final treatment (the room was unoccupied during that time). The plates were placed on three locations 1) on the patient’s chest, at 30 cm from the oral cavity; 2) next to the dental instruments on the unit; 3) at 150 cm from the patient’s oral cavity (Figure 1).

To determine the microbial load per cubic meter, the air was sampled actively using the BioSampler® (SKC Inc, Eighty Four, Pennsylvania, USA). The pump in the BioSampler® was calibrated with a rotameter (SKC Inc., Eighty Four, Pennsylvania, USA) according to manufacturer’s instruction, to maintain a flowrate of 12.5 L/min. Air was drawn into a 5 mL vessel with phosphate-buffered saline (PBS). PBS allows longer sampling time, less bacterial loss and 91% sampling efficiency compared to the use of sterile water [17–19]. The sampler was wrapped in an icepack to avoid liquid evaporation and overheating from the pump. The BioSampler® was placed left of the patient’s mouth, at 50 cm distance from the oral cavity and at 80 cm height from the floor. Sampling time was recorded to allow calculation of CFU/m^3^. Prior to the experiment, the BioSampler® was tested *in vitro* to determine the detection limit. The detection limit was determined at 100 CFU per plate/m^3^ for active sampling. After pilot testing with passive sampling, active sampling was only conducted during treatment.

Both the passive and active sampling experiments during patient treatment were performed 16 times per clinic during 4 to 5 days, depending on the schedule of the practitioner.

### Microbial contamination of dental unit water

Samples from DUWLs were taken from the air-water syringe of the dental units of every location on each sample day before the first treatment took place. The syringe was flushed for 30 seconds prior to sampling. A universal tube of 50 mL was filled with water, marked and stored at 4°C upon transportation to the laboratory.

### Laboratory analyses

Agar plates from passive sampling were incubated immediately upon arrival to the laboratory on the same day. The medium from the BioSampler® was vortexed for 30 seconds, 500 μL of medium was plated on blood agar plates in quadruplicate and incubated aerobically and anaerobically at 37°C. Another 500 μL was plated in duplicate onto R2A plates and incubated aerobically at 23°C.

The sampled DUWL water was vortexed for 30 seconds, 100 µl and 500 µl were plated on R2A agar in triplicate and plates were incubated aerobically at 23°C to determine the CFU/mL heterotrophic bacteria.

The number of colonies was counted after 7 days of incubation. Plates with more than 500 CFUs per plate were assigned as TTC; too many colonies to count. For the determination of the average CFU, plates with TTC were conservatively designated as 500 CFU/plate. All colonies of each plate were scraped off with a cotton swab, suspended in Eppendorf tubes containing 500 μL PBS. The aerobic, anaerobic and R2A samples were pooled per location and per time point and stored at −80°C for sequencing.

### Sequencing

For DNA isolation Eppendorf tubes with sample were thawed and centrifuged at 18,000 *g* for 5 minutes. Supernatant was removed and the pellet was resuspended with 200 µl of Tris-EDTA buffer. The supernatants were transferred to assigned wells in deep well plates and DNA was extracted and purified according to Volgenant et al [20]. The 16 S rRNA V4 gene amplicons were prepared according to Koopman et al. [21], with as exception that the 16 S rRNA gene amplification was done with 30 cycles. The final Amplicon was sequenced on an Illumina MiSeq system (Illumina, San Diego, CA, USA) using the MiSeq Reagent Kit V3 (2 x 251 nt) at the department of Core Facility Genomics, Amsterdam UMC. The flow cell was loaded with 10 pmol and 50% PhiX.

The paired-end reads were processing using USEARCH v.8.0.1623 [22]. Due to a collapse in quality towards the end of the reverse read, the reverse read was truncated to 150 nt. Next, the pairs were merged using a maximum expected error of 1 and a merged sequence length between 249 and 258 nt. The merged sequences were dereplicated and sorted by abundance using a minimum occurrence of 8.

These sequences were searched against the SILVA rDNA database v.132, 16 S rDNA sequences, clustered at 99% with majority consensus taxonomies produced by the QIIME team [23,24]. Searching was carried out with USEARCH (-usearch_local -strand plus -maxrejects 4096 – maxaccepts 256 – maxhits 50 – id 0.90 -query_cov 0.90). The USEARCH output was parsed to construct a 90% majority consensus taxonomic lineage of the (max.) 50 hits per query using a query coverage of ≥95% and sequence similarity of ≥95%. Finally, all merged sequences were searched against this dereplicated set (using USEARCH – search_exact) as to construct an ‘OTU’ table listing the counts of each unique sequence (instead of an OTU) in each sample [24].

### Data analyses

CFU counts were log transformed. The mean CFU values for both sampling methods were calculated for each location and time point. The Kruskal–Wallis test and the Mann–Whitney U test were applied to assess the differences in CFU counts. An abundance of <20 of each unique sequence was considered noise. Therefore, these sequences were removed. The number of taxa was counted per location/timepoint. The source of the taxa was assigned, either human or environmental/water, based on the best available evidence (see appendix 1).

The mean differences in number of taxa between clinics were analysed using the Kruskal–Wallis test followed by post-hoc analyses. The mean differences between all samples per clinic were analysed using the Friedman test. The mean difference between two samples was analysed using the Mann–Whitney U test. All values were adjusted using the Bonferroni correction for multiple testing. Correlations between CFU counts from different locations or samples were calculated using the Spearman correlation coefficient (Bonferroni correction for multiple testing). A p-value < 0.05 was considered statistically significant. All statistical analyses were conducted using SPSS Statistics for Windows (version 25.0, Armonk, NY, IBM Corp.).

## Results

### DUWL microbiology

On average, the dental unit water in clinic 1 consisted of 2.7 (SD 2.4) log_10_ CFU/mL, water from clinic 2 consisted of 3.4 (SD 3.1) log_10_ CFU/mL, and water from clinic 3 consisted of 3.6 (SD 3.5) log_10_ CFU/mL. No bacteria were recovered from water samples from clinic 4. The water samples contained between 2 and 6 different taxa (mean of 4.6 taxa per sample). The number of taxa in the water samples differed significantly between the clinics (Kruskal–Wallis test 6.93; p = 0.031). Water from clinic 2 contained significantly more taxa compared to water from clinic 1 (p = 0.009). The water samples contained mainly *Sphingomonas, Delftia* and *Caulobacter*, while *Pedobacter, Stenotrophomonas*, and *Pseudomonas* were identified only in the water of clinic 3. The number of DUWL bacteria correlated significantly with the number of water bacteria found on the R2A plates on the chest area (r = 0.56, p = 0.031).

DUWL from clinic 4 was disinfected using Anoxyl (250ppm chlorine, weekly) and Oxygenal (0.02% H_2_O_2_ continuously). DUWLs from clinics 1 and 3 were disinfected using Dentosept (0.0114% H_2_O_2_ continuously; 1.41% H_2_O_2_ weekly). And clinic 2 used a bacterial filter in the afferent waterline and weekly doses of Anoxyl (250ppm chlorine) as disinfectants for DUWL.

### Microbial load in the air, passive sampling

During sampling, the air-water syringe, ultrasonic scaler, air polisher and highspeed drills and handpieces were used while treating the patients, depending on the individual treatment plan. All clinics used a high-volume evacuator and a saliva ejector simultaneously. The mean CFU/plate is reported in Table 1. The average CFU per plate ranged from 0.1 to 2.8 log_10_. Lowest CFUs were found before and after treatment and at 150 cm distance from the head of the patient in all clinics. Therefore, 150 cm from the head of the patient was considered a control site for all passive air measurements.

**Table 1.** Mean (SD) Log_10_ of bacterial counts per growth method per location and moment

	Clinic 1			Clinic 2			Clinic 3			Clinic 4		
	mean CFU/plate (SD)			mean CFU/plate (SD)			mean CFU/plate (SD)			mean CFU/plate (SD)		
	Aerobe	Anaerobe	R2A	Aerobe	Anaerobe	R2A	Aerobe	Anaerobe	R2A	Aerobe	Anaerobe	R2A
**Passive sampling**												
*Time point*												
Before treatment	1.1 (0.5)	1.0 (0.7)	1.3 (1.1)	0.8 (0.5)	0.7 (0.7)	0.9 (0.9)	1.1 (0.9)	1.0 (1.0)	1.3 (1.0)	−0.1 (−0.4)	0.1 (−0.3)	0.1 (−0.3)
After treatment	1.1 (0.8)	0.8 (0.6)	1.0 (0.7)	1.0 (0.8)	0.9 (0.6)	1.1 (1.1)	1.4 (1.1)	1.3 (1.1)	1.3 (0.9)	0.5 (0.2)	−0.1 (−0.1)	0.2 (−0.3)
*Location (during treatment)*												
Chest 30 cm	2.8 (2.7) ^a,b^	2.8 (2.7) ^a,b^	2.6 (2.6) ^a,b^	2.4 (2.4) ^a,b^	2.1 (1.1) ^a,b^	1.4 (2.7) ^a,b^	2.7 (2.7) ^a,b^	2.6 (2.7) ^a,b^	2.7 (2.7) ^a,b^	1.2 (1.2)	1.4 (15)	0.5 (0.4)
Instrument plate	1.4 (1.1) ^a,b^	1.4 (1.0) ^a,b^	1.3 (0.9) ^a,b^	1.6 (1.9) ^a,b^	1.3 (1.0) ^a,b^	1.5 (1.9) ^a,b^	1.5 (1.9) ^a,b^	1.5 (1.9)^a,b^	1.4 (1.9) ^a,b^	0.3 (0.2)	0.7 (1.0)	−0.1 (0)
Control 150 cm	1.2 (1.0)	1.2 (1.1)	1.3 (1.0)	1.2 (0.9)	1.1 (0.9)	1.1 (0.9)	1.3 (1.3)	1.2 (1.3)	1.0 (0.9)	0.2 (−0.2)	0.2 (0.2)	0.1 (−0.1)
**Active sampling** *(during treatment)*												
	mean CFU/m^3^ (SD)			mean CFU/m^3^ (SD)			mean CFU/m^3^ (SD)			mean CFU/m^3^ (SD)		
50 cm from oral cavity	2.4 (2.7)	1.8 (2.2)	2.1 (2.5) ^a,b^	2.2 (2.0) ^a,b^	1.9 (2.0)	2.2 (2.0)	2.5 (2.5)^a,b^	1.9 (2.1)	2.6 (3.3)^a,b^	1.6 (1.4)	1.4 (1.6)	0 (0)

^a^Significant higher count compared to baseline.

^b^Significant higher count compared to follow up (Mann–Whitney U-test, p < 0.05).

Highest CFUs were retrieved from the chest and instrument area during treatment, and there were no significant differences between aerobic and anaerobic plates for these locations (p > 0.05). In contrast, before and after treatment, the aerobic counts were significantly higher than anaerobic counts (p < 0.05, Wilcoxon signed rank test on pairs). Also, at the control sites, the aerobic counts were higher compared to the anaerobic counts (p < 0.05, Wilcoxon signed rank test on pairs). The bacterial counts on the chest area were significantly higher compared to all other sampled locations (p < 0.001). The bacterial counts next to the instruments were significantly higher compared to before and after treatment, and the control location in all clinics (p < 0.01). The microbial load in the air of clinics 1, 2, and 3, as measured on the control locations 18 hours after the last treatment, was comparable (p > 0.05), but significantly higher compared to clinic 4 (p < 0.001).

### Microbial load in the air, active sampling

The mean CFU/m^3^ counts are reported in Table 1. The average CFU/m^3^ ranged from 0 to 2.6 log_10_. The microbial load in the air from clinics 1, 2, and 3 was significantly higher on R2A plates compared to clinic 4 (p < 0.05). Based on the aerobic counts, only clinic 2 and 3 had a significantly higher count compared to clinic 4 (p < 0.05). No significant difference was found on the microbial load in the air between all clinics on the anaerobic plates (p = 0.18).

### Microbial composition

The mean, modus, and ranges of the number of retrieved taxa per time point and per location are reported in Table 2. Bacteria from either human or water origin were found on all sampled locations and moments. A total of 52 human-derived taxa (oral and/or skin, HDT), and 36 water-derived taxa (WDT) were identified. The HDT *Actinomyces, Corynebacterium, Staphylococcus*, and *Streptococcus* and the WDT *Acinetobacter, Enhydrobacter*, and *Sphingomonas* were present in all sampled locations and at all moments. The number of HDT ranged from between 0 and 25 taxa per location or time point, and between 0 and 9 for WDT. An overview of taxa per location and time point is presented in Appendix 1. The clinics did not differ from each other in mean number of HDT (Kruskal–Wallis 7.80; p = 0.05), but there was a difference in locations and sample moments per clinic (Friedman test clinic 1: X^2^ = 12.81, p < 0.001; clinic 2: X^2^ = 5.32, p = 0.022; clinic 3: X^2^ = 11.52, p = 0.001; clinic 4: X^2^ = 9.52, p = 0.002). The sampling area on the chest had a higher number of taxa compared to before treatment (p = 0.014), and the control site (p = 0.028).

**Table 2.** Mean, modus and range of number of different taxa identified per sampling time or sample moment. Values are based on all clinics grouped together

	Before treatment	After treatment	Control	Chest	Instruments	Active samples
Human derived taxa (HDT)						
Mean	5	5	6.13^a^	9	6.64	4.97^a^
Modus	5	7	4	7	4	5
Range	1–9	0–10	1–25	1–21	1–15	2-11
Water derived taxa (WDT)						
Mean	2.1	2	2.5	2.7	2.57	1.43^a^
Modus	1	2	2	2	2	1
Range	1–4	0–7	0–6	0–7	0–6	0–9

* = significant higher number compared to chest sampled area (post-hoc analyses, Bonferroni corrected *p* < 0.05.

The number of WDT in air samples differed significantly between clinics (Kruskal–Wallis test 31.0; p < 0.001). The air from clinic 4 contained a significantly lower number of WDT taxa compared to the other clinics (no bacteria were retrieved from DUWL of clinic 4). No significant differences were found in number of taxa between different locations and sample moments (p > 0.05). The samples retrieved from active sampling at the patient’s chest had a significantly lower number of taxa compared to passive sampling (p < 0.001).

## Discussion

A large proportion of the air samples, from our study, that were taken before treatment, after treatment and during treatment at the control location did not meet the criteria for clean air (guidelines for clean air: SS EN ISO 14,698–1:2003). Only the university clinic met these criteria (<2 CFU/plate in 30 minute exposure time). Increased microbial air contamination was particularly found during treatment at the patient’s chest. This zone also contained the highest number of taxa, mainly from human origin. Close to the treatment site, aerobic bacteria were present in equal amounts to anaerobic bacteria. Locations further away from the patient contained significantly more aerobic bacteria than anaerobic bacteria, suggesting their origin is less likely to come from the dental treatment. The results of our study indicate that contamination of the aerosols from both human and water origin during treatment mostly settle in the close proximity of the head of the patient. This is in line with a previous study [25], although other studies reported no difference in microbial counts near the patient’s head and at further distance from the treatment zone [11,26].

The air in the university clinic contained significantly lower numbers of water-derived bacteria, as measured on R2A agar, compared to clinics 1–3. The air in clinic 2 and 3, both with the highest bacterial counts in the effluent DUWL water, had the highest microbial counts on R2A plates and the highest number of WDT during patient treatment. The university clinic, however, had the lowest contamination of the DUWL, low levels of DUWL in this clinic were reported previously [27,28]. The air ventilation rate in the university clinic was optimal, while it seemed suboptimal in the other clinics. Our results indicate that when infection control measures, such as good air ventilation and good control of microbial contamination of the DUWL, are optimal the spread of bacteria through aerosols is acceptably low.

So far, this is the first study to provide an assessment of the microbial composition of dental aerosols using next-generation sequencing. The microbial load of the air in the dental clinics turned out to be too low for reliable DNA-isolation and the subsequent sequencing procedure of the original samples. Thus, unfortunately, a cultivation step was needed to enable identification of the bacteria. Therefore, it was impossible to determine the relative number of taxa per sample. In addition, the culturing step introduced bias since all unculturable bacteria could not be counted/sequenced. Analyses were performed to genus level, since determination on species level using sequencing technique is less reliable. Moreover, more detailed information concerning species level was not needed to answer our research questions.

The obtained bacterial sequences were assigned a taxonomy on genus level and categorized into human-derived bacteria or water-derived bacteria. No distinction was made between oral and skin bacteria, since genera such as *Staphylococcus* and *Streptococcus* can be found in the oral cavity as well as on the skin. For infection control, it is not essential to make this distinction, because transmission of both oral bacteria and skin bacteria from the patient should be prevented.

As expected, a relatively high number of different human taxa were found on the chest area of the patient and on the instrument area, corresponding with a high number of CFU/plate on these locations. Human and water-derived bacteria were found in all samples, which indicates that droplet nuclei containing these bacteria remain present in the air of the clinic after they become airborne. However, the contamination level was low when no patient was treated in the room. *Staphylococci* were identified in all samples, which indicates human contamination [11,12,29].

The presented findings were consistent within each clinic, indicating that the two sampling methods used were reproducible. However, both methods missed part of the bacterial community. Only viable bacteria that can be cultured could be retrieved. The passive sampling method missed microorganisms that did not settle on the plates, that needed a specific growth medium or that became inactive when dispersed in the air [30]. The active sampling method missed microorganisms because a culturing step was necessary. Thus, the results from our study are an underrepresentation of the actual contamination rate.

In the light of the current situation of a pandemic with the SARS-CoV-2 virus, the data from this study show that dental aerosols, containing bacteria from human origin, will be distributed around the head of the patient during treatment. We should, however, keep in mind that in our study, only bacterial contamination was determined. Viruses are much smaller and therefore they can probably reach greater distances from their source. So, contamination of the air with viruses via droplet nuclei, can probably reach further than was found for bacteria in our study. Furthermore, settling of bacteria was determined, which is defined as the number of bacterial cells that are settled on a certain surface. When studying the transmission of airborne microorganisms, it is more important to study how long these microorganisms remain airborne. Studies have proven that viruses, such as the bacteriophage MS2, can remain airborne for hours in or on small aerosol particles. Since normal settling only reduces the viral load for about 48% in 45 minutes, it is advised to maintain proper ventilation of the treatment room to remove smaller aerosol particles that may contain viruses, such as SARS-CoV-2 [31].

In conclusion, the bacterial contamination caused by droplets and droplet nuclei is only of concern during dental treatment and tends to concentrate around the head of the patient. No increase in bacterial contamination was found at 1.5 m from the oral cavity. Both human- and water-derived bacteria were found throughout the treatment room. These results stress the importance of infection control measures on surfaces in close proximity to the head of the patient. Adequate air ventilation and a low level of contamination of DUWLs likely lead to lower levels of microbial contamination of the air in dental clinics.

## Appendix 1

**Table:**

**Bacterial genera identified as originating from the human microbiome (oral; skin) per sample**											
**30 min before first treatment**											
*Actinomyces*		*Aerococcus*			*Arthrobacter*				*Bacillus*		*Brevibacterium*
*Capnotytophaga*		*Clostridium*			*Corynebacterium*				*Dermabacter*		*Dermacoccus*
*Dietza*		*Enterococcus*			*Fastidiospilia*				*Kocuria*		*Micrococcus*
*Nesterenkonia*		*Pantoea*			*Paracoccus*				*Rhodococcus*		*Rothia*
*Staphylococcus*		*Streptococcus*									
**30 min after end of treatment**											
*Actinomyces*		*Aerococcus*			*Arthrobacter*				*Bacillus*		*Brachybacterium*
*Brevibacterium*		*Clostridium*			*Corynebacterium*				*Dermabacter*		*Dermacoccus*
*Fastidiospilia*		*Frigoribacterium*			*Gordonia*				*Granulicatella*		*Kocuria*
*Macrococcus*		*Micrococcus*			*Nesterenkonia*				*Paracoccus*		*Rhodococcus*
*Selenomonas*		*Staphylococcus*			*Streptococcus*				*Veillonella*		
**During treatment, at 150 cm from head of the patient**											
*Actinomyces*		*Aerococcus*			*Anaeroglobus*				*Arthrobacter*		*Bacillus*
*Brachybacterium*		*Brevibacterium*			*Campylobacter*				*Corynebacterium*		*Dermacoccus*
*Dialister*		*Dietza*			*Eikenella*				*Fastidiospilia*		*Fusobacterium*
*Gemella*		*Granulicatella*			*Kocuria*				*Lactobacillus*		*Macrococcus*
*Micrococcus*		*Neisseria*			*Nesterenkonia*				*Pantoea*		*Paracoccus*
*Parvimonas*		*Peptostreptococcus*			*Prevotella*				*Prevotella*		*Serratia*
*Staphylococcus*		*Streptococcus*			*Veillonella*						
**During treatment, on chest area**											
*Actinomyces*		*Aerococcus*			*Anaeroglobus*				*Arthrobacter*		*Bacillus*
*Bifidobacteria*		*Brachybacterium*			*Brevibacterium*				*Campylobacter*		*Capnocytophaga*
*Corynebacterium*		*Dermabacter*			*Dermacoccus*				*Dialister*		*Eikenella*
*Enterobacter*		*Enterococcus*			*Fastidiospilia*				*Frigoribacterium*		*Fusobacterium*
*Gemella*		*Granulicatella*			*Haemophilus*				*Kocuria*		*Lactobacillus*
*Megasphaeara*		*Micrococcus*			*Negativicoccus*				*Neisseria*		*Neomicrococcus*
*Nesterenkonia*		*Oribacterium*			*Paeniglutamicibacter*				*Pantoea*		*Paracoccus*
*Parvimonas*		*Pasteurellaceae*			*Peptostreptococcus*				*Prevotella*		*Rhodococcus*
*Selenomonas*		*Staphylococcus*			*Stomatobaculum*				*Streptococcus*		*Veillonella*
**During treatment, instrument area**											
*Aerococcus*		*Anaeroglobus*			*Arthrobacter*				*Bacillus*		*Bifidobacteria*
*Brachybacterium*		*Brevibacterium*			*Campylobacter*				*Capnocytophaga*		*Corynebacterium*
*Dermabacter*		*Dermacoccus*			*Dialister*				*Eikenella*		*Enterobacter*
*Enterococcus*		*Fastidiospilia*			*Frigoribacterium*				*Fusobacterium*		*Gemella*
*Granulicatella*		*Haemophilus*			*Kocuria*				*Lactobacillus*		*Megasphaeara*
*Micrococcus*		*Negativicoccus*			*Neisseria*				*Neomicrococcus*		*Nesterenkonia*
*Oribacterium*		*Paeniglutamicibacter*			*Pantoea*				*Paracoccus*		*Parvimonas*
*Pasteurellaceae*		*Peptostreptococcus*			*Prevotella*				*Rhodococcus*		*Selenomonas*
*Staphylococcus*		*Stomatobaculum*			*Streptococcus*				*Veillonella*		
**During treatment, active sampling**											
*Actinomyces*	*Aerococcus*			*Arthrobacter*				*Bacillus*			*Brachybacterium*
*Corynebacterium*	*Dermabacter*			*Dermacoccus*				*Dolosigranlum*			*Enterobacter*
*Enterococcus*	*Fastidiospilia*			*Gemella*				*Gordonia*			*Granulicatella*
*Kocuria*	*Micrococcus*			*Neisseria*				*Nesterenkonia*			*Pantoea*
*Paracoccus*	*Rhodococcus*			*Serratia*				*Staphylococcus*			*Streptococcus*
*Streptomyces*	*Veillonella*										
**Bacterial genera identified as originating from the water microbiome per sample**											
**30 min before first treatment**											
*Acinetobacter*			*Brevundimonas*			*Caulobacter*	*Cutibacterium*			*Enhydrobacter*	
*Moraxella*			*Sphingomonas*			*Sporosarcina*	*Virgibacillus*				
**30 min after end of treatment**											
*Acinetobacter*			*Bacilus*			*Brevibacillus*	*Chryseobacterium*			*Cutibacterium*	
*Enhydrobacter*			*Finegoldia*			*Hymenobacter*	*Roseomonas*			*Sphingomonas*	
*Sporosarcina*											
**During treatment, at 150 cm from the head of the patient**											
*Acinetobacter*			*Brevibacillus*			*Brevidumonas*	*Chryseobacterium*			*Cutibacterium*	
*Deinococcus*			*Delftia*			*Enhydrobacter*	*Finegoldia*			*Flavobacterium*	
*Hymenobacter*			*Massilia*			*Paenibacillus*	*Pseudomonas*			*Psychrobacter*	
*Roseomonas*			*Solibacillus*			*Sphingomonas*	*Stenotrophomonas*			*Virgibacillus*	
**During treatment, on chest area**											
*Acinetobacter*			*Caulobacter*			*Chryseobacterium*	*Delftia*			*Enhydrobacter*	
*Finegoldia*			*Hymenobacter*			*Lysinibacillus*	*Massilia*			*Pedobacter*	
*Pseudomonas*			*Roseomonas*			*Sphingomonas*	*Sporosarcina*			*Stenotrophomonas*	
**During treatment, instrument area**											
*Acinetobacter*			*Anaerococcus*			*Brevibacillus*	*Brevudimonas*			*Chryseobacterium*	
*Cutibacterium*			*Delftia*			*Enhydrobacter*	*Fictibacillus*			*Hymenobacter*	
*Lysinibacillus*			*Lysobacter*			*Paenibacillus*	*Pedobacter*			*Promicromonospora*	
*Pseudomonas*			*Psychrobacter*			*Roseomonas*	*Solibacillus*			*Sphingomonas*	
*Stenotrophomonas*											
**During treatment, active sampling**											
*Acinetobacter*			*Brevudimonas*			*Chryseobacterium*	*Cutibacterium*			*Elizabethkingia*	
*Enhydrobacter*			*Exiguobacterium*			*Finegoldia*	*Hymenobacter*			*Paenibacillus*	
*Pseudomonas*			*Pseudoxanthomonas*			*Roseomonas*	*Sphingomonas*			*Stenotrophomonas*	
*Virgibacillus*											
**Dental unit waterline effluent water**											
*Bosea*			*Brevudimonas*			*Caulobacter*	*Delftia*			*Elizabethkingia*	
*Flavobacterium*			*Methylobacterium*			*Sphingomonas*	*Stenotrophomonas*				
